# PAK4 interacts with p85 alpha: implications for pancreatic cancer cell migration

**DOI:** 10.1038/srep42575

**Published:** 2017-02-16

**Authors:** Helen King, Kiruthikah Thillai, Andrew Whale, Prabhu Arumugam, Hesham Eldaly, Hemant M. Kocher, Claire M. Wells

**Affiliations:** 1 Division of Cancer Studies, King’s College London, UK; 2 Barts Cancer Institute, a CRUK centre of Excellance, Queen Mary University of London, UK; 3 Dept of Haematopathology Oncology Diagnostic Service, Addenbrooke’s Hospital, Cambridge, UK

## Abstract

It has been reported that p21-activated kinase 4 (PAK4) is amplified in pancreatic cancer tissue. PAK4 is a member of the PAK family of serine/threonine kinases, which act as effectors for several small GTPases, and has been specifically identified to function downstream of HGF-mediated c-Met activation in a PI3K dependent manner. However, the functionality of PAK4 in pancreatic cancer and the contribution made by HGF signalling to pancreatic cancer cell motility remain to be elucidated. We now find that elevated PAK4 expression is coincident with increased expression levels of c-Met and the p85α subunit of PI3K. Furthermore, we demonstrate that pancreatic cancer cells have a specific motility response to HGF both in 2D and 3D physiomimetic organotypic assays; which can be suppressed by inhibition of PI3K. Significantly, we report a specific interaction between PAK4 and p85α and find that PAK4 deficient cells exhibit a reduction in Akt phosphorylation downstream of HGF signalling. These results implicate a novel role for PAK4 within the PI3K pathway via interaction with p85α. Thus, PAK4 could be an essential player in PDAC progression representing an interesting therapeutic opportunity.

Pancreatic ductal adenocarcinoma (PDAC) is highly aggressive. It is one of the most lethal solid malignancies and has a 5-year survival rate of less the 3%. The *K-RAS* gene is frequently mutated in PDAC^1^,^2^,^3^. Within PDAC, it is believed that there are three main effector pathways downstream of K-RAS; these are the mitogen activated protein kinase (MAPK), phosphatidylinositol-3-Kinase (PI3K) and RalGEF pathways. Interestingly *PAK4* gene amplification has also been reported in PDAC and associated with K-RAS mutation status^4^,^5^,^6^. PAK4 is a member of the PAK family of serine/threonine kinases which act as effectors for several small GTPases. They are involved in a wide range of signalling pathways including cell motility, survival and proliferation; therefore, abnormal PAK signalling can contribute to a number of disease states^7^. In particular, PAK4 is oncogenic when overexpressed, promoting cell survival, migration and anchorage-independent growth^8^. It has been established that PAK4 may be a driver of pancreatic cancer cell migration^5^. While the mode of PAK4 regulation is not well understood, there is evidence from our lab^9^, and others, that PAK4 may lie within a phosphatidylinositol-3-Kinase (PI3K) pathway^10^. However, a direct *in cellulo* relationship between PAK4 and RAS has not been reported and the nature of the relationship between PAK4 and PI3K remains to be fully elucidated.

Among the different oncogenic K-RAS activated effector pathways that are involved in PDAC, the PI3K pathway is a key mediator of RAS-driven oncogenesis and is emerging as one of the most critical^1^; it has been estimated that approximately 50% of cancers have deregulation of this pathway involved in their tumourigenesis^11^,^12^. PI3K signalling leads to the activation of Akt, which is a known indicator of aggressiveness in PDAC^13^,^14^,^15^ and correlates with outcome^16^,^17^.

Typically the PI3K/AKT pathway has been considered primarily to be responsible for survival signalling and proliferation, and Akt has recently been identified as a central signalling component during pancreatic tumourigenesis^18^. However there is accumulating evidence to suggest that Akt signalling also directly contributes to cellular motility^19^. PI3K is also activated through association with the c-Met receptor. c-Met acts as a high affinity receptor for HGF, which is also known as scatter factor^20^. HGF/c-Met signalling has been associated with pancreatic tumorigenesis^21^,^22^ where a marked increase in c-Met expression was observed in PDAC tumour samples and increased levels of circulating HGF were reported in pancreatic cancer patients^23^. Moreover, transwell and scattering assays^24^,^25^,^26^ report a response to HGF however direct visualisation and cell migration speeds have not been reported.

## Results

### Expression of PAK family kinases in pancreatic cancer cell lines

Previous studies of pancreatic cancer had not investigated the expression profile of all PAK family members in pancreatic cancer nor established how PAK expression correlated with expression levels of the PI3K:RAS axis. We therefore sought to compare expression between pancreatic cancer cell lines and normal controls. Two epithelial cell lines were used: HPDE cells which are a human papillomavirus (HPV)−16 E6E7 immortalised cell line derived from normal adult pancreatic tissue^27^ and DechTERT cells, which are primary cells collected and hTERT immortalised^28^. Three cancer cell lines were used. Capan1 cells are a well differentiated, colony forming cell line which was sourced from a liver metastasis, with mutations in *KRAS, TP53, INK4A, SMAD4* and *BRCA2*^29^. PaTu8988S and PaTu8988T cells were isolated from a liver metastasis of a primary pancreatic adenocarcinoma and carry mutations in *KRAS* and *TP53* with methylation of the 5’ CpG island of *INK4A*^30^. PaTu8988S/T are also reported to have a PAK4 amplification^31^. Of the two lines PaTu8988T is the most poorly differentiated and invasive line^30^. Initially we validated the epithelial status of HPDE and dechTERT cells based on E-cadherin expression and junctional localisation. We found that neither cell line exhibited junctional E-cadherin (Figure S1A) to address this issue in HPDE cells we stimulated junctional formation via incubation with calcium prior to expression analysis (Figure S1B). dechTERT did not express E-cadherin (Figure S1C) and thus we were unable to use calcium treatment with these cells. However it is known that pancreatic duct epithelial cells express cytokeratins (CK) 7, 8, 18 and 19 and we observed high expression of CK18 (Figure S1D) therefore we retained these cells in our analysis. Of our cancer cell lines the two colony forming cell lines exhibited high levels of E-Cadherin expression (Figure S1C).

**Figure 1:**
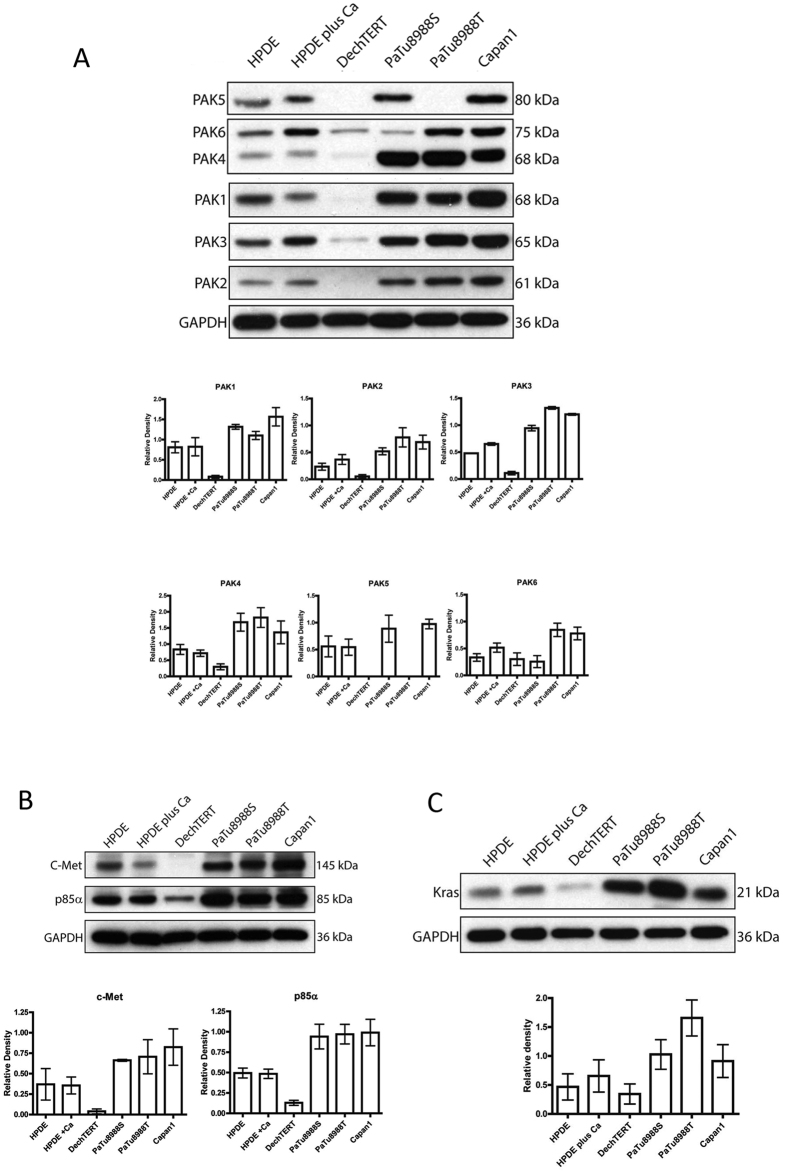
Expression levels of the PAK4:RAS:PI3K pathway in pancreatic cancer cells. (**A**) Expression of PAK family proteins. Cell lysates (as indicated) were probed for expression of PAK1–6 using isoform specific antibodies. Expression levels were quantified by densitometry after normalising relative expression to the loading control (GAPDH). (**B**) Expression of c-Met and p85α in pancreatic cell lines. Cell lysates (as indicated) were probed for expression of C-Met and p85alpha. Expression levels were quantified by densitometry after normalising relative expression to the loading control (GAPDH). (**C**) Expression of K-RAS. Cell lysates (as indicated) were probed for expression of K-RAS using an isoform specific antibody. Expression levels were quantified by densitometry after normalising relative expression to the loading control (GAPDH). In all cases membranes were cut before probing and blots have been cropped to size. All blots are representative of three independent experiments.

Having established the underlying provenance of our cell line panel we proceeded to investigate the expression of PAK family kinases. We found that all family members could be detected (using isoform specific antibodies) in pancreatic cancer cells (Fig. 1A). Of the PAK family members PAK4 was consistently expressed at higher levels in pancreatic cancer cell lines compared to normal controls. Moreover, we were also able to detect PAK4 expression using an isoform specific antibody in human PDAC tissue (Figure S1E). These data suggest a focus on PAK4 in pancreatic cancer would be appropriate. Using the same cell lines we were also able to establish that expression levels of KRAS, c-Met and the p85 alpha subunit of PI3K closely mirror the profile of PAK4 (Fig. 1B and C).

### Pancreatic cancer cell lines exhibit a response to HGF

**Figure 2:**
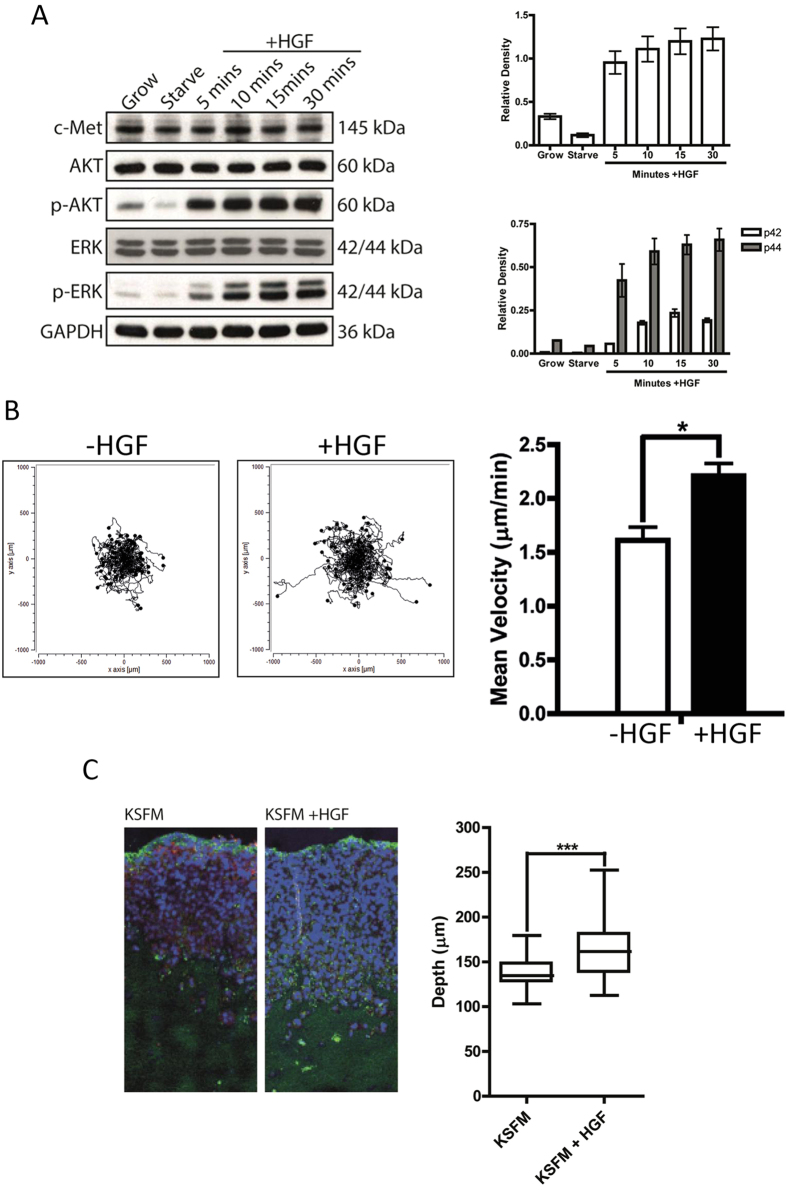
Pancreatic cancer cells have a biochemical and migratory response to HGF. (**A**) PaTu8988T cells were maintained in growth conditions or serum starved overnight before being stimulated with HGF for the time indicated. Lysates of treated and untreated cells were separated by SDS-PAGE and probed for c-Met and phosphorylated proteins as indicated, lysates were then re-probed for total protein level and GAPDH as a loading control. Blots are representative of three independent experiments. Each experimental repeat was individually quantified using ImageJ and represented in graphical form after normalising to the loading control (GAPDH). In all cases membranes were cut before probing and blots have been cropped to size. All blots are representative of three independent experiments. (**B**) PaTu8988T cells were serum starved overnight, stimulated by HGF and filmed for 16 hr with time-lapse video microscopy. n = 60 individual cells per condition were tracked over 3 separate experiments. The mean migration speed ± SEM calculated for each condition. *p < 0.05. (**C**) An Organotypic co-culture of PaTu8988T and PS-1 cells were fed with either KSFM or KSFM + HGF (see materials and methods for details of model) every 2 days for 14 days. Gels were harvested fixed, processed and stained for DAPI (blue) and pan-cytokeratin (green). Bar = 100 um. To measure cancer cell invasion the depth of invasion was measured at multiple points along the images from three independent experiments. ***p < 0.0005. Representative image is shown.

We have previously shown that PAK4 is activated downstream of HGF^9^. HGF stimulated migration has been reported to occur in Rat pancreatic cells^32^ but few human pancreatic cancer cell lines have been directly tested for migration speed. We now demonstrate that human pancreatic cancer cells elicit both an intracellular signalling (Fig. 2A) and migratory response to HGF stimulation (Fig. 2B and Figure S2A). Moreover using a physiomimetic organotypic invasion assay^33^ we demonstrate that PaTu8988T cell invasion is promoted by HGF (Fig. 2C). However, we did not find any evidence that PAK4 autophosphorylation levels are modulated by HGF in pancreatic cancer cells (Figure S2B). This is perhaps not unexpected as recent studies suggest that in a number of cell types, PAK4 is constitutively autophosphorylated^34^.

### PAK4 is required for pancreatic cancer cell migration in response to HGF

**Figure 3:**
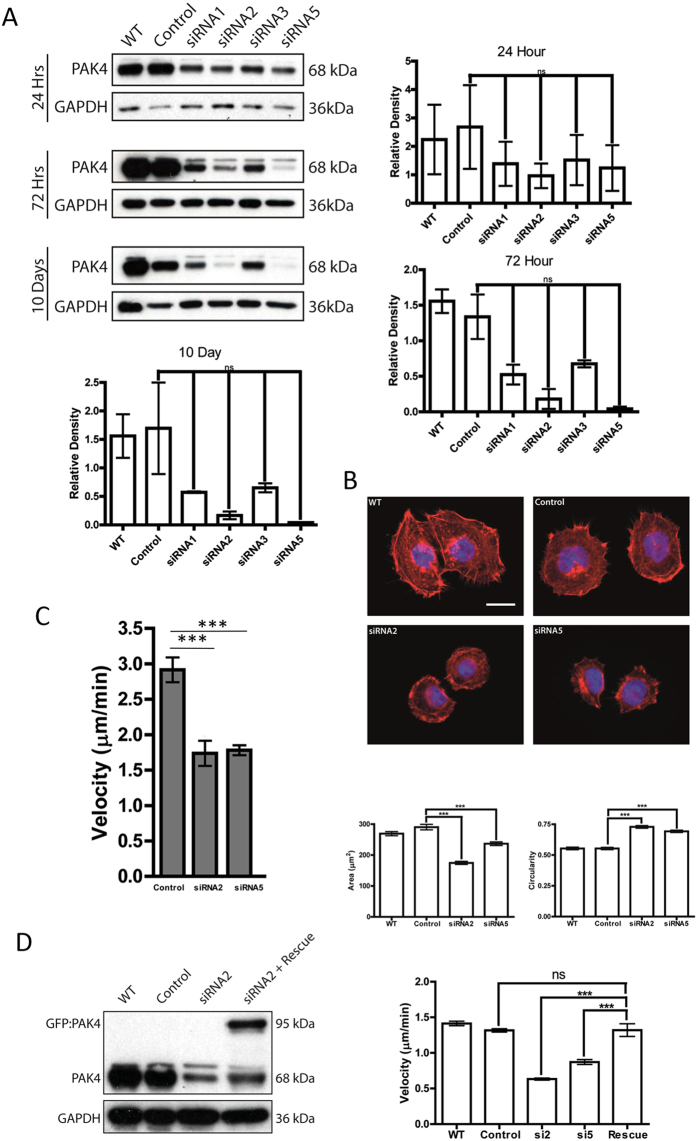
Depletion of PAK4 expression suppresses pancreatic cancer cell migration response to HGF. **(A)** PaTu8988T cells were treated with control or four different siRNA oligos targeted to PAK4. Depletion compared to sicontrol using siRNA2 and siRNA5 could be detected up to 10 days following treatment. Expression levels were quantified by densitometry after normalising relative expression to the loading control (GAPDH). In all cases membranes were cut before probing and blots have been cropped to size. All blots are representative of three independent experiments. **(B)** PaTu8988T cells were untreated (WT) or treated with control or siRNA2 or siRNA5 oligos targeted to PAK4. Cells were seeded onto collagen coverslips and stained for F-actin. Images of 60 cells for each condition over three separate experiments were quantified using ImageJ software to calculate cells area and circularity. Bar = 10 um **(C)** PaTu8988T cells were treated with control or siRNA2 or siRNA5 oligos targeted to PAK4. Cells were seeded on collagen coated plates. serum starved overnight and stimulated with HGF and filmed for 16 hr with time-lapse video microscopy. n = 60 individual cells per condition were tracked over 3 separate experiments. The mean migration speed ± SEM calculated for each condition. ***p < 0.0005. **(D)** PaTu8988T cells were treated with control or siRNA2 oligo targeted to PAK4 and then transfected with GFP-siRNA resistant PAK4 where indicated. Cells were filmed for 16 hr with time-lapse video microscopy. n = 15 individual cells per condition were tracked over 3 separate experiments. The mean migration speed ± SEM calculated for each condition. ***p < 0.0005.

**Figure 4:**
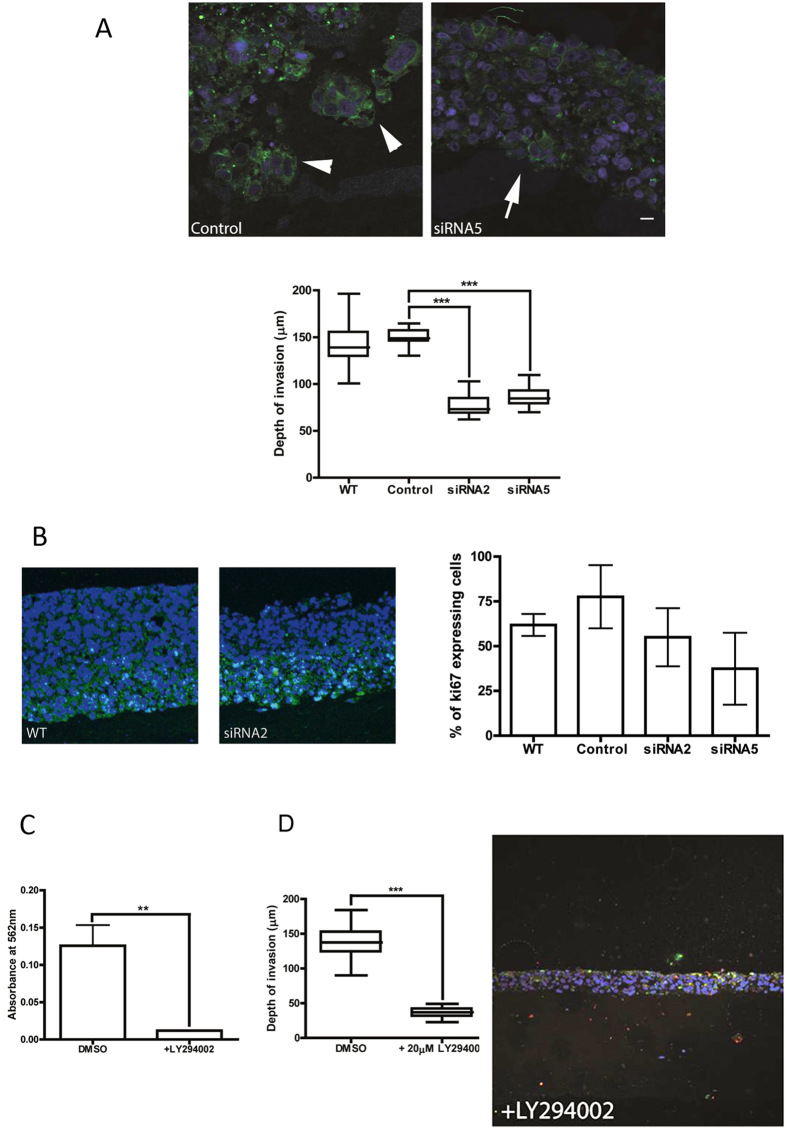
Depletion of PAK4 expression suppresses HGF-stimulated pancreatic cancer cell invasion. **(A)** An Organotypic co-cultures of sicontrol or siRNA5 treated PaTu8988T cells and PS-1 cells were fed with KSFM + HGF (see materials and methods for details) every 2 days for 14 days. Gels were harvested fixed, processed and stained for DAPI (blue) and pan-cytokeratin (green). Bar = 100 um. To measure cancer cell invasion the depth of invasion was measured at multiple points along the images from three independent experiments. ***p < 0.0005. Representative image is shown. Arrow heads indicate single and clusters of invasive cells. Arrow highlights lack of clusters/individual cell invasion. **(B)** An Organotypic co-culture of sicontrol or siRNA2 treated PaTu8988T cells and PS-1 cells were fed with KSFM + HGF (see materials and methods for details of model) every 2 days for 14 days. Gels were harvested fixed, processed and stained for DAPI (blue) and ki67 (green). The % of ki67 positive cells was calculated for multiple images from three independent experiments. ***p < 0.0005. Representative image is shown. Bar = 100 um **(C)** PaTu8988T cells were treated with LY294002 or DMSO control and monitored for impact on cell proliferation using an MTT assay. **(D)** An Organotypic co-culture of DMSO or LY294002 treated PaTu8988T cells and PS-1 cells were fed with KSFM + HGF (see materials and methods for details) every 2 days for 14 days. Gels were harvested fixed, processed and stained for DAPI (blue) and pan-cytokeratin (green). To measure cancer cell invasion the depth of invasion was measured at multiple points along the images from three independent experiments. ***p < 0.0005. Representative image is shown. Bar = 100 um.

Having established that PAK4 is expressed in pancreatic cancer cells and that those cells with high expression of PAK4 also have a migratory response to HGF we sought to establish whether PAK4 expression was required for the migratory response to HGF. PAK4 was depleted from PaTu8988T cells as these cells perform well in both the 2D and 3D migration assays (Fig. 2B and C) using siRNA technology. Two separate oligonucleotides were identified (siRNA2 and siRNA5) as capable of specifically depleting PAK4 expression up to 10 days post treatment (Fig. 3A). We have previously reported two PAK4 depletion phenotypes: reduced cell area^35^ or increased levels of cell adhesion^36^,^37^. Detailed analysis of the PAK4 depletion in PaTu8988T cells suggests that these cells follow the cell phenotype where a reduced cell area is observed (Fig. 3B) but no indication of increased cell adhesions (data not shown). PAK4 depleted cells with a reduced area were found to exhibit a cell migration defect^38^ and reduced migration has been reported in pancreatic cancer cells when PAK4 expression is depleted^5^. Consistent with previous reports we found that PAK4 depleted PaTu8988T cells have a significant reduction in mean cell migration speed (Fig. 3C). Moreover, re-expression of siRNA resistant PAK4 in these cells was able to rescue the cell migration deficiency (Fig. 3D). Importantly we also found that reduced PAK4 expression significantly reduced the level of cancer cell invasion in the HGF-mediated organotypic invasion assay (Fig. 4A and Figure S2C). Whilst our 2D migration assay is not impacted by changes in the rate of proliferation performance in the organotypic assay can be influenced by changes in proliferation rate. Given that previous reports suggest that depletion of PAK4 can reduce proliferation rate^7^ we tested our knockdown cells in an MTT proliferation assay. We found that PAK4 depleted cells had a modest reduction in cell proliferation rate (Figure S2D). We then tested whether the reduction in cell proliferation was the source of reduced cell invasion by staining the organotypic assay for cleaved caspase 3 and ki67. We found that there was little difference in the cleaved caspase 3 signal between control and knockdown cells (Figure S2E). We also found that although the level of ki67 positive staining was reduced in the PAK4 depleted assays this was not significant (Fig. 4B) and could not fully account for the difference in invasion potential detected between these two populations. Indeed, DAPI positive nuclei staining suggested that in PAK4 depleted assays the cells are more compacted in the upper layer (less space between positive nuclei) suggesting that absolute number of cells is not dramatically reduced (Fig. 4B and Figure S2E) and thus there is not a large proliferation defect.

### PI3K activity is required for HGF-mediated pancreatic cancer invasion

Our results had suggested that PAK4 activity is required for 2D migration and 3D invasion mediated via HGF signalling (Fig. 3C and Fig. 4A). Our previous work demonstrated that PAK4 activity can be suppressed by incubation with the PI3K inhibitor LY294002^9^. We therefore tested whether the PI3K pathway also influences pancreatic cancer cell migration mediated by HGF. Incubation of pancreatic cancer cells with 20 nM LY294002 had a significant effect on cell proliferation (Fig. 4C) and a dramatic effect on invasion potential (Fig. 4D) which is almost certainly mainly attributable to a loss of cell proliferation. However we could not detect any cancer cells invading the underlying matrix suggesting that inhibition of PI3K activity may also suppresses invasion (Fig. 4D).

### PAK4 binds to p85alpha via the proline rich domain

**Figure 5:**
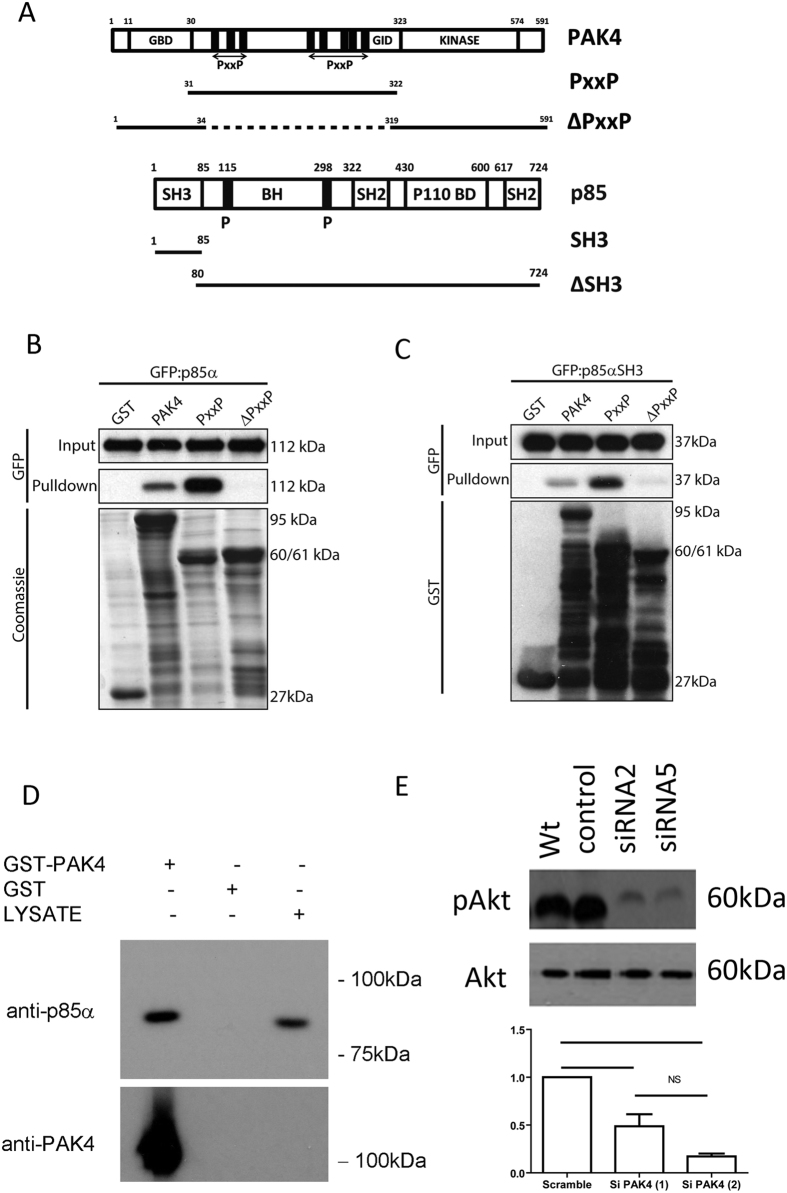
PAK4 interacts with p85 via the SH3 domain **(A)** schematic illustrating the structure of full length and domain mutants utilised here. **(B)** HEK293T cells expressing GFP-p85alpha were lysed and the lysates used in a GST pulldown with GST-PAK4 or PAK4 derivates as bait. The GST pulldown was probed for the presence of GFP-p85alpha using an anti-GFP antibody. Coommasie stain illustrates GST proteins. **(C)** HEK293T cells expressing GFP-p85alpha-SH3 domain alone were lysed and the lysates used in a GST pulldown with GST-PAK4 or PAK4 derivates as bait. The GST pulldown was probed for the presence of GFP-p85alpha-SH3 domain using an anti-GFP antibody. The blot was re-probed for GST. GST alone was used as a control. **(D)** PaTu8988T cell lysates were incubated with GST or GST-PAK4 and the GST-pull down probed for endogenous p85alpha. PaTu8988T lysate was used a positive control for antibody signal. **(E)** Wt, sicontrol, siRNA2 and siRNA5 treated (72 h) PaTu8988T cells were serum starved overnight and then stimulated with HGF for 15 minutes. Cells were lysed and lysates probed for the level of Akt phosphorylation (S473). Samples were re-probed for total Akt. Levels of pAkt (S473) were quantified by densitometry after normalising relative expression to total Akt. In all cases membranes were cut before probing and blots have been cropped to size. All blots are representative of three independent experiments.

Our data suggests that both PAK4 and PI3K have functionality during the migration of pancreatic cancer cells in the presence of HGF. However, there are no reports in the literature that directly link PAK4 and PI3K. Interestingly protein profile predictions (ScanProsite) suggested that N-terminal regions of PAK4 might interact with the PI3K regulatory subunit p85α. We therefore tested whether there might be an interaction between PAK4 and p85α. We used expression in HEK293T cells for our structure function analysis as this system yields high levels of protein expression. Initially we generated two PAK4 mutants one expressing the proline rich region of PAK4 (PAK4PxxP comprising amino acids 31–322) the other expressing PAK4 with the PXXP region deleted (PAK4ΔPxxP comprising amino acids 1–34 plus 319–591). (Fig. 5A schematic). We then tested whether GST, GST tagged full length PAK4 or GST tagged PAK4PxxP or GST tagged PAK4ΔPxxP could pulldown GFP tagged full length p85α (Fig. 5B). We found that PAK4 binds to p85α via the PXXP region in PAK4. Such binding would suggest it is mediated via an SH3 domain interaction. p85α has an N-terminal SH3 domain. In order to investigate this, two domain mutants of p85α were created (Fig. 5A schematic) alongside full-length p85α; one mutant consists of just the SH3 domain (comprising amino acids 1–85) and one mutant is missing the SH3 domain (ΔSH3, comprising amino acids 80–724). These were tagged with GFP and transfected into HEK293 cells prior to their use in a PAK4 GST pulldown. The SH3 mutant was shown to bind to full length PAK4 and to the PxxP construct, but not to the ΔPxxP construct (Fig. 5C) as would be expected if PAK4 is interacting with the p85α via its SH3 domain. In contrast, when using the ΔSH3 construct, no binding could be observed between any of the PAK4 domain mutants (Figure S3A). This further supports evidence for the SH3 domain being vital in the interaction between PAK4 and p85α. Importantly, we also detect an interaction between PAK4 and endogenous p85α in the pancreatic cancer cells (Fig. 5D).

### PAK4 is required for maximal Akt phosphorylation in pancreatic cancer cells

Our data suggests that both PAK4 and PI3K are essential components of the migratory response of pancreatic cancer cells to HGF both in 2D and 3D. Moreover we can now report a direct interaction between PAK4 and the PI3K complex.

Since p85α is binding to PAK4 outside of the kinase domain we would predict that p85α is not a PAK4 substrate and indeed we find no evidence that p85α is phosphorylated in the presence of PAK4 (Figure S3B) nor can we find any evidence that the presence of p85α can promote PAK4 autophosphorylation or substrate phosphorylation activity (Figure S3B). Thus it is likely that the interaction between PAK4 and p85α is focussed on regulation of PI3K activity perhaps by modulating the inhibitory interaction between p85α and the p110 catalytic subunit^39^. To test this hypothesis we monitored the level of Akt phosphorylation in PAK4 depleted cells as a readout of PI3K activity. In support of our hypothesis we found that phosphorylation of Akt was significantly reduced in PAK4 depleted cells cultured either in the presence of serum (Figure S3C) or more importantly when specifically stimulated with HGF (Fig. 5E).

## Discussion

In this study we have demonstrated that the highly invasive pancreatic cancer cell line, PaTu8988T, exhibits a biochemical and migratory response to HGF stimulation. Moreover, we find that PI3K signalling and PAK4 expression are both required for optimal response to HGF both in 2D and 3D invasion assays. Importantly, PAK4 is overexpressed in a range of pancreatic cancer cell lines (including PaTu8988T), which correlates with an increased expression of c-Met, K-ras and the p85α subunit of PI3K. Furthermore, we have identified a link between PAK4 and components of the Ras:PI3K pathway which converges on phosphorylation of Akt. Taken together our study supports further investigation of PAK4:PI3K signalling nexus in pancreatic cancer as a possible therapeutic target.

siRNA-mediated depletion of PAK4 was shown to reduce both 2D migration and 3D invasion, which may be through regulation of the downstream effector protein, Akt; these data have also been shown to be phenocopied through pharmacological inhibition of PI3K. Thus, PAK4 may be involved in the PI3K pathway to promote pancreatic cell invasion. There is already considerable evidence to suggest a role for PAK4 in metastasis and invasion in other cell types and links between PAK4 and pancreatic cancer have been previously reported^4^,^5^,^6^. PAK4 is the most widely studied of the group II PAKs and has been shown to contribute significantly to cancer cell invasion in gastric cancer^40^, glioma^41^, choriocarcinoma^42^ and prostate^35^,^38^ among others, both *in vitro* and *in vivo*. PAK4 is able to promote cancer cell invasion through modulation of the actin cytoskeleton and microtubules. It is known that this regulation occurs through interaction with various proteins including Cdc42, which leads to reorganization of actin in the formation of filopodia^43^; GEF-H1 mediated interaction with microtubules allows for increased motility^44^; phosphorylation of LIMK by PAK4 enables inhibition of the actin filament disassembly protein, cofilin^38^,^45^ and association with the scaffold protein Gab1 leads to modulation of cell migration within lamellipodia^46^. However, how PAK4 may drive pancreatic cancer cell invasion has yet to be fully elucidated. Genomic studies have shown that PAK4 may be associated with oncogenic K-Ras, which is mutated in over 90% of pancreatic tumour samples^6^ and *in vitro* cell-based assays have shown that shRNAmediated knockdown of PAK4 in a pancreatic cancer cell line reduced cell migration^5^. Data presented here demonstrate that PAK4 is able to interact with the p85α subunit of PI3K. This novel interaction between PAK4 and p85α was found to be dependent on the proline rich region of PAK4 and the SH3 domain of p85α. It has previously been suggested that an interaction between PAK4 and an SH3 domain containing protein could mediate kinase activity^47^. We did not detect any global changes in activity when PAK4 was incubated with p85 but further studies would be warranted to test substrate specificity.

Further to our novel interaction studies we also demonstrated that depletion of PAK4 expression led to a significant loss of Akt phosphorylation. These findings, have been recently corroborated in other tissue types where a reduction in PAK4 in both NIH3T3, gastric cancer and cells lines resulted in a reduction of Akt at Ser473^10^,^48^,^49^ In addition, this phenotype was seen to be rescued by expression of constitutively active K-RAS^48^. This further supports the hypothesis of a RAS/PI3K/PAK4/AKT pathway, but the comprehensive mechanisms behind PAK4/Akt interplay still remain unclear. Whether PAK4 affects phosphorylation of Akt at Thr308, as well as Ser473, has not yet been investigated. The regulatory p85 subunit of PI3K serves three main roles: stabilisation, negative regulation and recruitment^39^. To initiate PI3K signalling, in response to HGF, the p85 subunit associates with the c-Met receptor. This interaction can either be direct, whereby the p85 subunit associates with phosphorylated tyrosine residues via its SH2 domain, or indirectly via the scaffold protein Gab1^50^. After association p85a and the phosphorylated tyrosine residues takes place, p85-mediated inhibition of the catalytic p110 subunit is relieved enabling p110 to transfer phosphate groups to initiate downstream signalling after being brought into contact with their lipid substrates at the cell membrane^51^. Our data would suggest that PAK4 does not phosphorylate p85α, thus the interplay between PAK4, p85α and Akt phosphorylation remains to be fully elucidated. It might however be hypothesised that PAK4 binding to p85α somehow relives p110 inhibition downstream of HGF. Alternatively, PAK4 depletion from cells may indirectly influence Akt phosphorylation levels by leading to increased expression of negative regulators of the PI3K pathway or increased expression/activation of AKT phosphatases.

Alternatively the modulation of Akt may be more direct. Akt is recruited to the membrane by binding of its PH domain to the PI3K generated PIP3, and this translocation to the membrane is crucial for Akt activation^52^. It is known that both PDK1 and the mTORC2 complex^52^ impact on AKT activity through phosphorylation at Thr308 and Ser473, respectively, downstream of PI3K. It has been shown that PDK1 can interact with PAK1^53^. Indeed, in NIH-3T3 cells, activated PAK1 increased phosphorylation of Akt at both sites, which was shown to be independent of PAK1 kinase activity. Despite differences in sequence homology, PAK1 and PAK4 share a number of substrates^7^, so it could therefore be hypothesised that PAK4 affects the phosphorylation of Akt downstream of PI3K activation by a similar mechanism. Further investigation will be required to elucidate the molecular mechanism involved.

Although typically the PI3K/AKT pathways has been considered primarily to be responsible for survival signalling and proliferation, there is accumulating evidence to suggest that Akt signalling contributes to cellular motility, including in metastatic cancer cells^19^. Indeed, phosphorylation of Akt at both Thr308 and Ser473 was required for motility of lung endothelial cells downstream of HGF, with pharmacological inhibition of PI3K via LY294002 treatment leading to reduced phosphorylation at both sites and decreased lamellipodia formation^54^. Furthermore, it has recently been reported that the Wnt family member, Wnt5A, promotes phosphorylation of Akt at Ser473 downstream of PI3K to promote osteosarcoma cell migration^55^, supporting the hypothesis that PAK4 regulation of Akt phosphorylation at Ser473 may also be required for PDAC cell migration. Whilst a role for Akt in pancreatic cancer cell migration has not been previously documented; results presented here provide a strong case for future investigation. Given that there is considerable pharmacological interest in PAK, PI3K and Akt inhibition a greater understanding of how this signalling nexus mediates pancreatic cancer cell invasion is of primary importance.

## Materials and Methods

### Antibodies and reagents

Unless indicated, primary antibodies were used at a dilution of 1:1000 for Western blotting. Anti-GAPDH was purchased from Millipore and used at a dilution of 1:20000. Rabbit anti-PAK1, rabbit anti-phospho-PAK4 (Ser 474)/PAK5 (Ser 602)/ PAK6 (Ser 560) and rabbit anti-PAK4, which also recognizes PAK6^35^ were purchased from Cell Signaling Technology. Rabbit polyclonal PAK4 specific antibody (raised against PAK4 peptide sequence CRRAGPEKRPKSSREG) has been described elsewhere^37^. Mouse anti-GFP was obtained from Roche. Rabbit anti-HA (Y-11) and mouse c-Myc (9E10) were purchased from Santa Cruz. HRP-conjugated secondary antibodies were purchased from DAKO and diluted 1:2000.

### DNA constructs and transfection

cDNA of PAK4, PAK4r (containing silent, siRNA refractory mutations), PAK4PXXP, PAK4DPXXP, p85alpha, p85 alphaSH3 and p85alphaDSH3 were cloned into pDONR207 using BP Gateway^®^ recombination to generate entry vectors as previously described^37^,^38^. PAK4/p85alpha derivatives were then transferred into either mammalian GFP/mRFP- or bacterial GST- expression destination vectors using LR Gateway^®^ recombination. HEK293 cells were transfected by calcium phosphate transfection according to manufacturers protocol (Sigma Aldrich). PaTu8988T cells were transfected using X-tremeGENE according to manufacturers protocol (Sigma Aldrich).

### Cell culture

PaTu8988T (kind gift of Dr. Frank Ulrich Weiß, Department of Medicine, Ernst Moritz Arndt Universität Greifswald, Greifswald, Germany), HEK293 (ATCC) and DechTERT cells were maintained in Dulbecco’s modified Eagle’s media (DMEM) supplemented with 10% v/v fetal bovine serum (FBS) and 1 mM penicillin/streptomycin. Capan1 cells were maintained in Roswell Park Memorial Institute-1640 (RPMI-1640) media supplemented with 10% v/v FBS with 1 mM penicillin/streptomycin. HPDE cells were maintained in keratinocyte serum-free media (KSFM) supplemented with 5ng/ml epidermal growth factor (EGF), 0.1 mg/ml bovine pituitary extract (BPE) and 1 mM penicillin/streptomycin. Pancreatic stellate cells (PS-1 cells) were maintained in DMEM:F12, supplemented with 10% v/v FBS, 1 mM penicillin/streptomycin and 1 μg/ml puromycin as a selection agent. All medium and reagents supplied by Sigma Aldrich UK except KSFM supplied by Life Technologies UK.

### Organotypic Culture Model

The organotypic gel matrix was prepared with a 1:1 ratio of collagen and matrigel. To this gel was added 1 volume 10xDMEM, 1 volume PS1-cell suspension, 1 voume foetal calf serum. The gel components were mixed thoroughly and neutralized with 0.1 M sodium hydroxide (NaOH). Gel was added to a 24-well tissue culture plate. The plate was then placed in an incubator for 1 hour at 37 °C until the gel had polymerized. After this, 1 ml of DMEM with 10% FBS was added on top of each gel before being returned to the incubator and left overnight. A cell suspension was prepared containing 5 × 105 PaTu8988T cells mixed with 2.5 × 105 PS-1 cells per gel in 1 ml DMEM containing 10% FBS. Media was aspirated carefully from on top of each gel and 1 ml of cell suspension then added dropwise to the centre of the gel. The gels were incubated at room temperature for 5 minutes to allow the cells to settle before being returned to the incubator and maintained at 37 °C for 48 hours. On day 4, the organotypic gels were raised onto steel grids to create an air-liquid interface. Onto each grid, a collagen gel-coated nylon sheet was carefully placed. The well of the plate was then filled with medium until it reached the undersurface of the grid. This was then classed as day 1 of the organotypic invasion assay. The gels were then returned to the incubator and maintained at 37 °C. The culture media was replaced every two days and the gels were harvested after 14 days of culture for paraffin embedding. For staining the sections were de-waxed and rehydrated prior to staining. Slides were de-waxed using xylene twice for 5 minutes each time. To rehydrate the sections an alcohol gradient was used. Each wash was incubated at room temperature for 2 minutes. Samples were then soaked in PBS (with calcium and magnesium). Antigen retrieval was performed using 0.01 M tri-sodium citrate (dihydrate) at pH6 under boiling conditions for 10 minutes. Following antigen retrieval slides were dried quickly. The sections were then permeabilised by 0.2% TritonX-100 in PBS for 5 minutes at room temperature. Sections were washed twice in PBS and then quenched in a 1 mg/ml sodium borohydride/PBS solution for 10 minutes. Sections were then washed twice in PBS before being blocked for 30 minutes at room temperature. A blocking solution containing 2% BSA, 0.02% fish skin gelatin and 10% FBS was used. Sections were then incubated overnight at 4 °C in the dark with the appropriate primary antibody. Sections were stained with anti-pan-cytokeratin and anti-smooth muscle actin (anti-SMA). The following day, sections were washed three times with PBS before being incubated for 1 hour at room temperature in the dark with the appropriate secondary antibody, followed by 10 minutes with DAPI. The sections were washed another three times in PBS and twice in ddH20 and mounted on slides. Images were collected on a Carl Zeiss LSM510 META laser scanning confocal microscope or a Nikon Eclipse Ti-E inverted A1R Si confocal microscope.

### siRNA transfection

Transient knockdown of PAK4 was achieved using human PAK4 siRNA oligonucleotide 2 (Qiagen; cat. no. S102660315; sequence CGAGAATGTGGTGGAGATGTA) and oligonucleotide 5 (Dharmacon; cat. no. D-003615–05; sequence GGGTGAAGCTGTCAGACTT). Control and *PAK4* specific oligonucleotides were added to cells using HiPerFect Transfection Reagent (Qiagen) according to the manufacturer’s instructions to a final concentration of 30 nM. Efficiency of knockdown was assessed by Western blotting after 48 h.

### Immunofluorescence and image analysis

Cells were seeded onto collagen I-coated coverslips. Following transfection or incubation overnight, cells were fixed in 4% paraformaldehyde in PBS for 20 min at RT and subsequently permeabilised with 0.2% Triton X-100 in PBS for 5 min. For F-actin staining, cells were incubated with either TRITC- or Alexa fluor 488-conjugated phalloidin (Invitrogen) diluted in PBS for 1 h at RT. Following this incubation, cells were washed 3 times in PBS. For detection of paxillin, primary antibodies were diluted in PBS with 3% bovine serum albumin (Fisher Scientific) and incubated for 2 h at RT. Following labeling with the primary antibody, cells were washed 3 times with PBS before incubation with either Alexa fluor 568 or 488-conjugated secondary antibodies (Invitrogen) and phalloidin. Cells were then imaged using an Olympus IX71 microscope with a 40X/NA 1.3 UPlanFLN oil-immersion objective and Image-Pro Plus software (supplied by MAG, UK).

### Time-lapse microscopy

Cells were plated onto collagen I-coated 6-well plates to which 25 mM Hepes was added. Immediately prior to filming, cells were treated with 10 ng/ml HGF to induce motility^56^. Each plate was placed on the automated heated stage of an Olympus IX71 microscope set at 37 °C and imaged with a 10X/NA 0.3 UPlanFLN objective lens. Images were collected using a Retiga SRV CCD camera, taking a frame every 5 min for 18 h from each of the wells using Image-Pro Plus software. Subsequently all the acquired time-lapse sequences were displayed as a movie and cells were tracked for the whole of the time-lapse sequence using Motion Analysis software (Andor Technology, Belfast, UK). This resulted in the generation of a sequence of position co-ordinates relating to each cell in each frame. At least 60 cells were tracked over 3 separate films for each experimental condition. Mathematical analysis was then carried out using Mathematica 6.0™ notebooks developed in house by Graham Dunn and Gareth E. Jones^38^. Statistical significance was accepted for P ≤ 0.05.

### GST-tagged protein purification and pulldown assays

GST proteins were purified from BL21-A1 cells (Invitrogen). Briefly, bacterial cells were transformed with pDEST15-GST-PAK4, or GST-PAK4 derivative expression vectors and cultured in LB broth supplemented with 100 μg/ml ampicillin until OD_600_ 0.4–0.6. Recombinant protein synthesis was induced overnight at 20 °C with 0.2% L-arabinose. Bacterial pellets were lysed in PBS containing complete mini protease inhibitor tablet (Roche) followed by sonication and centrifugation at 15,000 × g for 10 min at 4 °C to remove cell debris. The supernatant was then incubated with pre-washed Glutathione Sepharose 4B beads (GE Healthcare) for 2 h at 4 °C and the GST-fusion protein coupled beads were collected by centrifugation, washed three times and stored in 50% glycerol, 20 mM Tris-HCl pH 7.6, 100 mM NaCl and 1 mM DTT.

### Kinase assay

GFP expressing proteins were purified for use in the kinase assay using GFP-TRAP Chromotek, Germany according to the manufacturer’s instructions. Purified GST proteins were incubated in the presence or absence of GFP-purified proteins in kinase buffer (50 mM Tris-HCL pH7.5, 10 mM MgCl_2_ and 1 mM DTT) containing 30 μM ATP and 3 μCi of [γ^−32^P]ATP together with Histone H1 (Roche) for 30 min at 30 °C. The reaction was stopped by adding SDS loading buffer.

### Statistical analyses

Data sets were compared using two-tailed Students’ t-tests (unless otherwise stated in the figure legend) and presented as mean ± SEM. Statistical significance was accepted for p ≤ 0.05.

## Additional Information

**How to cite this article:** King, H. *et al*. PAK4 interacts with p85 alpha: implications for pancreatic cancer cell migration. *Sci. Rep.* **7**, 42575; doi: 10.1038/srep42575 (2017).

**Publisher's note:** Springer Nature remains neutral with regard to jurisdictional claims in published maps and institutional affiliations.

## Supplementary Material

Supplementary Figures
