# Supplementary material for: PAK4 interacts with p85 alpha: implications for pancreatic cancer cell migration
Source: Sci Rep. 2017 Feb 16;7:42575. doi: 10.1038/srep42575 (PMC5312077; doi:10.1038/srep42575)
Supplement: Supplementary Figures [file srep42575-s1.pdf]

# PAK4 interacts with p85 alpha:

# implications for pancreatic cancer cell migration

Helen King, Kiruthikah Thillai, Andrew Whale, Prabhu Arumugam, Hesham Eldaly,

Hemant M Kocher and Claire M Wells

Supplemental Figures

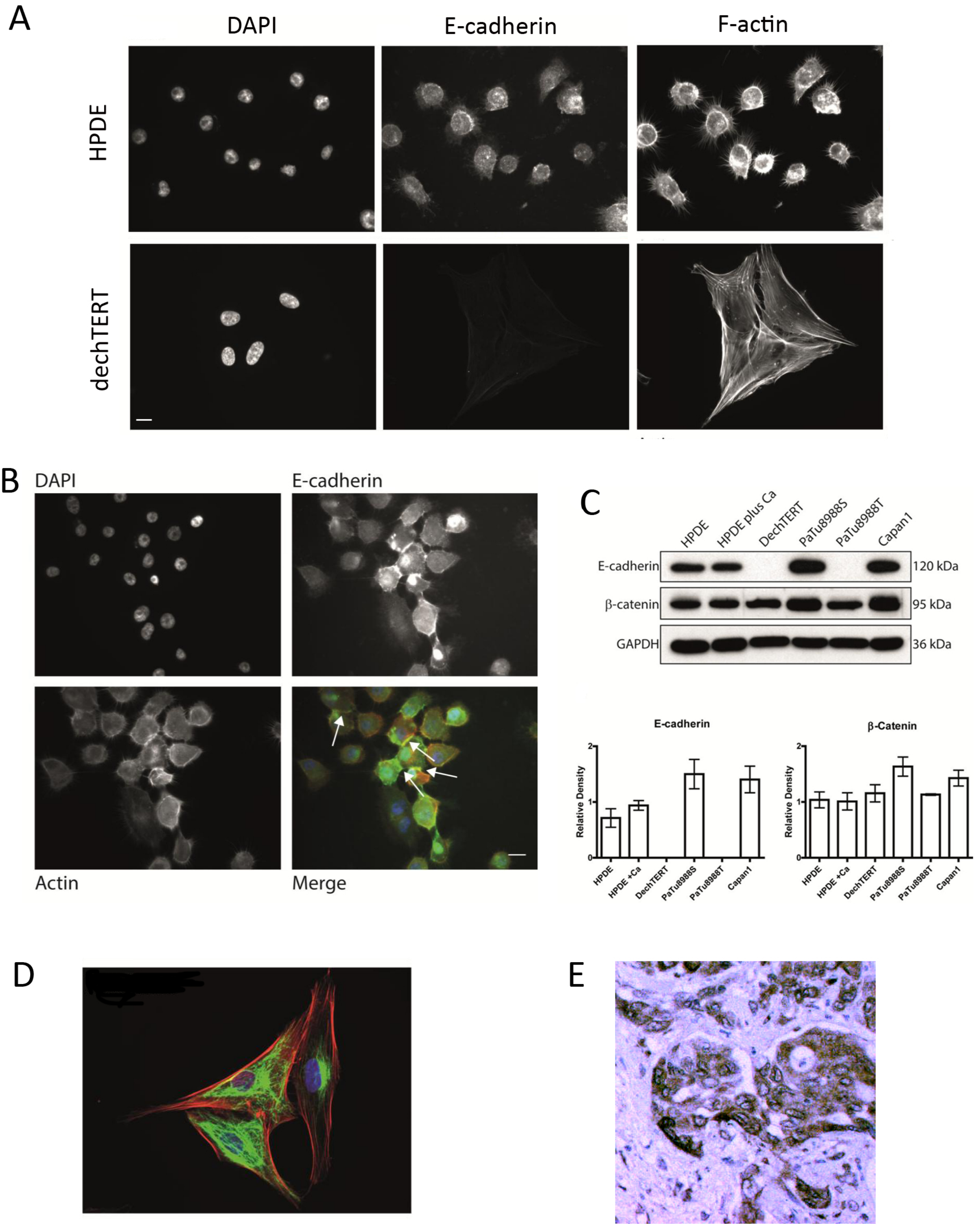
A
DAPI
E-cadherin
F-actin
HPDE
dechTERT
B
DAPI
E-cadherin
Actin
Merge
C
HPDE
HPDE plus Ca
DechTERT
PaTu8988S
PaTu8988T
Capan1
E-cadherin
β-catenin
GAPDH
120 kDa
95 kDa
36 kDa
E-cadherin
β-Catenin
Relative Density
2
1
0
HPDE
HPDE +Ca
DechTERT
PaTu8988S
PaTu8988T
Capan1
D
E

Figure S1

Figure S2

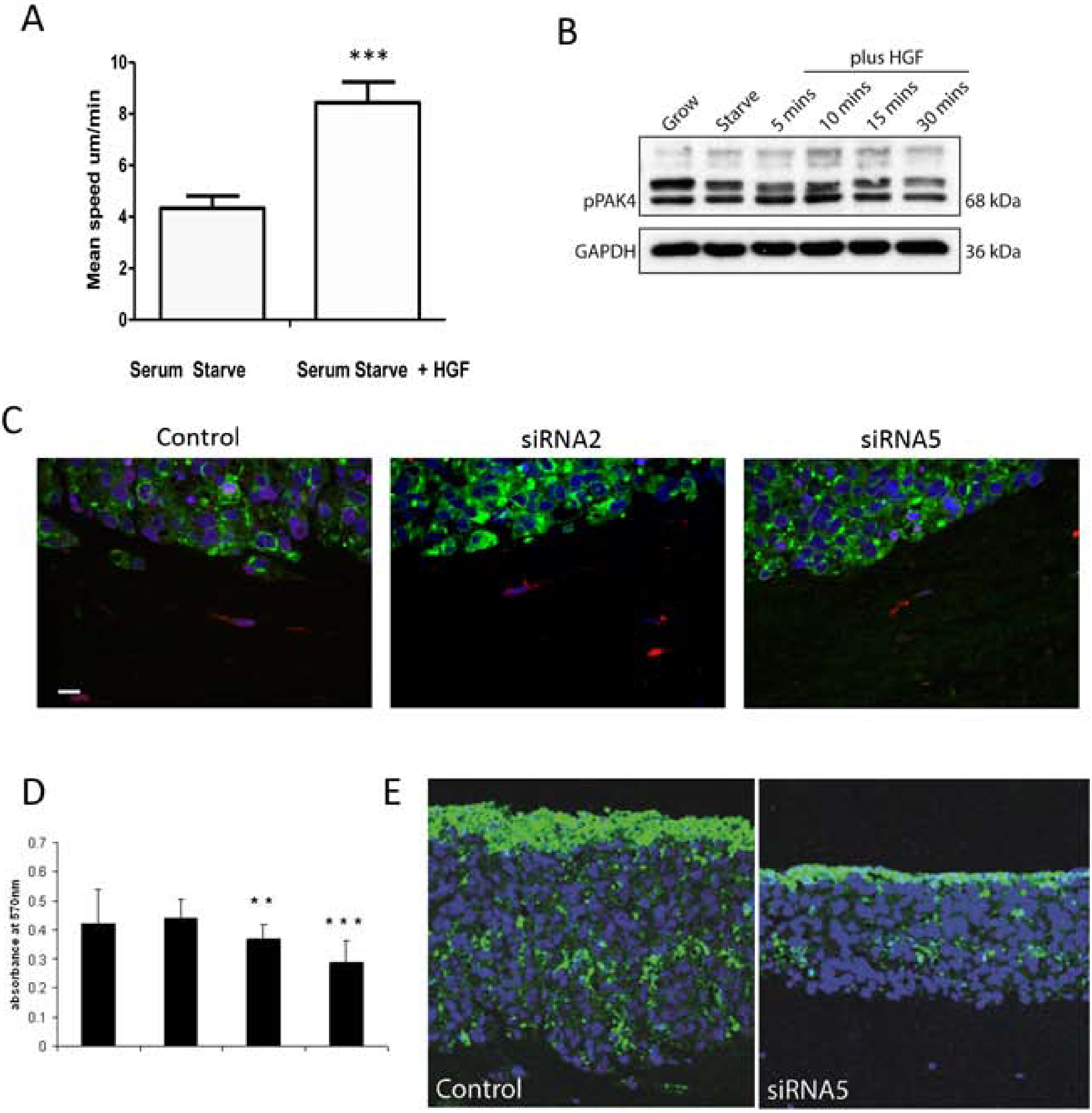

A
***
Mean speed um/min
10
8
6
4
2
0
Serum Starve
Serum Starve + HGF
B
plus HGF
Grow
Starve
5 mins
10 mins
15 mins
30 mins
pPAK4
68 kDa
GAPDH
36 kDa
C
Control
siRNA2
siRNA5
D
absorbance at 570nm
0.7
0.6
0.5
0.4
0.3
0.2
0.1
0
* *
* * *
E
Control
siRNA5

Figure S3

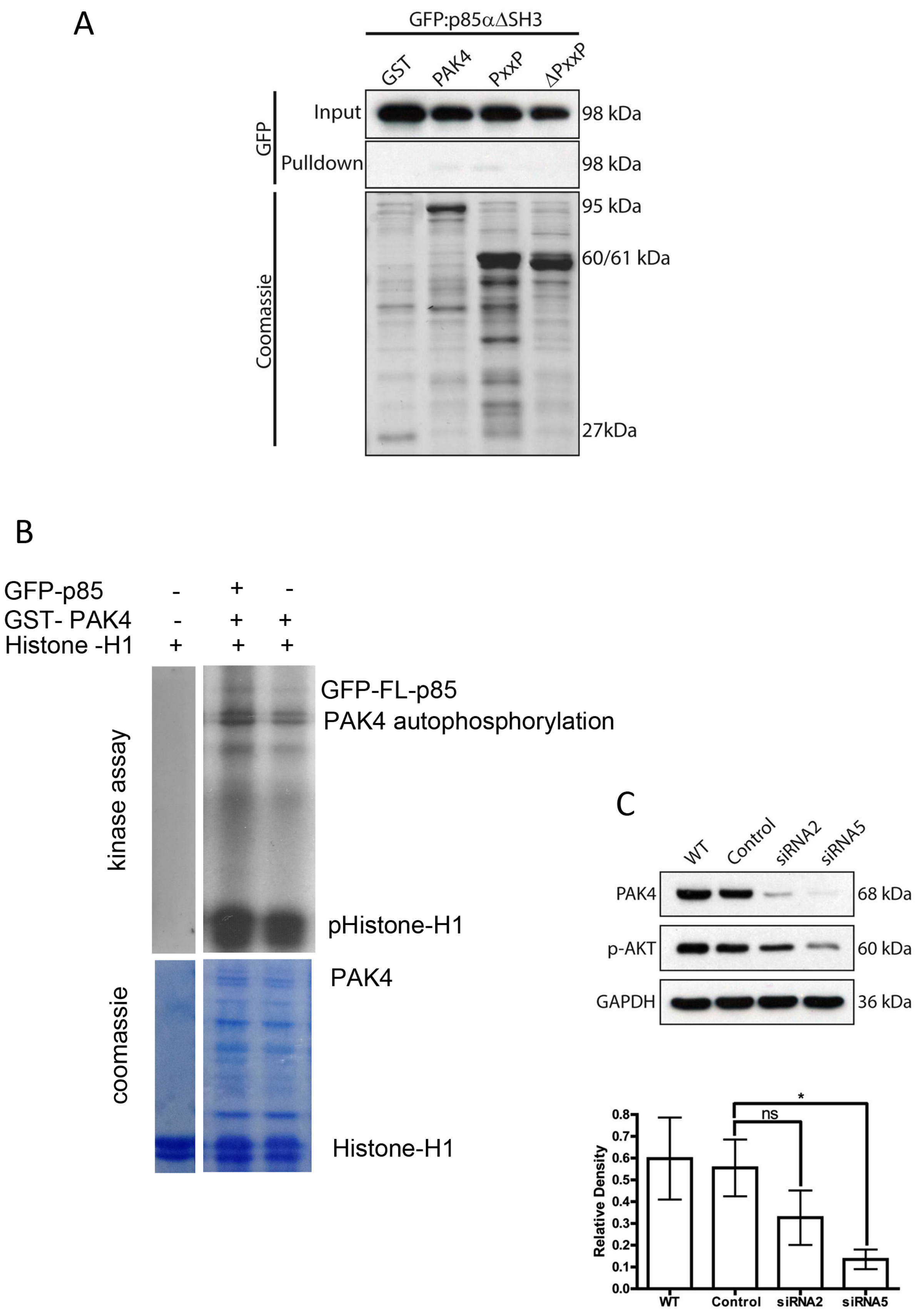

A
GFP:p85αΔSH3
GST
PAK4
PxxP
ΔPxxP
GFP
Input
Pulldown
98 kDa
98 kDa
Coomassie
95 kDa
60/61 kDa
27kDa
B
GFP-p85 - + -
GST- PAK4 - + +
Histone -H1 + + +
kinase assay
GFP-FL-p85
PAK4 autophosphorylation
pHistone-H1
coomassie
PAK4
Histone-H1
C
WT
Control
siRNA2
siRNA5
PAK4
68 kDa
p-AKT
60 kDa
GAPDH
36 kDa
Relative Density
0.8
0.7
0.6
0.5
0.4
0.3
0.2
0.1
0.0
ns
*
WT
Control
siRNA2
siRNA5

**Figure S1**

**(A)** HPDE and dechTERT cells were seeded on collagen coated coverslips and stained for DAPI, E-cadherin and F-actin Bar = 10um **(B)** HPDE cells were seeded on coverslips in the presence of calcium nitrate (1g/L) fixed and stained for DAPI, E-cadherin and F-actin. Arrow indicates cell: cell junction formation. Bar = 10um **(C)** Lysates from cell lines indicated were probed for E-cadherin, β-catenin and GAPDH as a loading control. Expression levels were quantified by densitometry after normalising relative expression to GAPDH. Blots are representative of three independent experiments. **(D)** DechTERT cells were seeded on collagen coated coverslips and stained for DAPI (blue), F-actin (red) and cytokerratin 18 (green). Bar = 10um **(E)** Immunohistochemical staining of human pancreatic cancer tissue. Samples stained for PAK4 (brown) using an in-house PAK4 specific antibody and counterstained haematoxylin (blue) to reveal cellular structure. Magnification is 400x.

**Figure S2**

**(A)** Capan-1 cells were seeded on collagen I, serum starved overnight and then stimulated with HGF. Cells were filmed for 16 hr with time-lapse video microscopy. n = at least 30 individual cells per condition were tracked over 3 separate experiments. The mean migration speed ± SEM calculated for each condition. *** $p<0.0005$

**(B)** PaTu8988T cells were maintained in growth conditions or serum starved overnight before being stimulated with HGF for the time indicated. Lysates of treated and untreated cells were separated by SDS-PAGE and western blotted for phospho-PAK4 and GAPDH as a loading control. Blots are representative of three

independent experiments. **(C)** Additional images of experiment depicted in Figure 4A illustrating lack of invasive cells in PAK4 depleted background. Prepared as Figure 4A stained for DAPI (blue) pan –cytokerratin (green) and smooth muscle actin (red). **(D)** MTT proliferation assay for WT, sicontrol (c), siRNA2 (si2) and siRNA5 (si5) treated cells. Cells were seeded at same density 48h post siRNA treatment and absorbance was measured 3 days post seeding. **(E)** DAPI (blue) and Caspase 3 staining (green) of sicontrol and siRNA5 treated cells following completion of organotypic assay.

**Figure S3**

**(A)** HEK293T cells expressing GFP-p85alphadeltaSH3 were lysed and the lysates used in a GST pulldown with GST-PAK4 or PAK4 derivates as bait. The GST pulldown was probed for the presence of GFP-p85alphadeltaSH3 using an anti-GFP antibody. Coommasie stain illustrates GST proteins. **(B)** In vitro kinase assay using GST-PAK4 in the absence and presence of GFP-p85. Top panel ia an auotradiograph bottom panel is a coomassie stain of gel –note PAK4 autophosphorylation and histone H1 phosphorylation but no indication of p85 phosphorylation. **(C)** PaTu8988T cells were untreated or treated with sicontrol, siRNA2 or siRNA5 oligos and maintained in growth conditions for 72 h. Lysates of treated and untreated cells were separated by SDS-PAGE and western blotted for PAK4, phospho-AKt (S473) and GAPDH as a loading control. Levels of pAkt (S473) were quantified by densitometry after normalising relative expression to total GAPDH. Blots are representative of three independent experiments
